# HIV Disclosure and Low HIV Stigma in a Gay Serodiscordant Couple: An Interpretative Phenomenological Case Study of the Discloser-Confidant Dynamics

**DOI:** 10.7759/cureus.74597

**Published:** 2024-11-27

**Authors:** Florian Thanasko, Maria Nikoloudi, Konstantina Antoniadou Anemi, Vassilis Kouloulias, Kyriaki Mystakidou

**Affiliations:** 1 Department of Radiology, Pain Relief and Palliative Care Unit, Aretaeio Hospital/National and Kapodistrian University of Athens School of Medicine, Athens, GRC; 2 Department of Developmental Psychology, National and Kapodistrian University of Athens School of Medicine, Athens, GRC; 3 Department of Radiology, Radiotherapy Unit, Attikon Hospital/National and Kapodistrian University of Athens School of Medicine, Athens, GRC

**Keywords:** discloser-confidant, hiv disclosure, hiv stigma, interpretative phenomenological analysis, serodiscordant gay couple

## Abstract

Introduction

HIV stigma levels are high in Greece. HIV stigma hinders testing, healthcare access, and treatment adherence, often leading to non-disclosure. The discloser navigates challenges by balancing the confidant's potential reactions, ranging from rejection and discrimination to the benefits of increased intimacy and liking. However, much research focuses on either the confidant’s reactions or the discloser’s role, leaving gaps in understanding the full disclosure process.

Methods

This qualitative case study explores how HIV disclosure, stigma, and trust-building unfold, focusing on the timing, reasons, and impact of disclosing HIV-positive status on the relationship dynamics between the discloser and the confidant. Semi-structured interviews were conducted with a serodiscordant gay couple, recruited from a hospital in Greece.

Results

Through interpretative phenomenological analysis, we identified a Superordinate Theme: The HIV Disclosure Process with three sub-themes: (1) Navigating HIV Disclosure: Decision Making and the Disclosure Event, (2) HIV Acceptance and Stigma: Pathways to Disclosure, and (3) HIV Disclosure: Building a Foundation of Trust and Navigating Life’s Broader Uncertainties. Investigator triangulation was used, enhancing the credibility and reliability of the findings.

Conclusion

Our case study explores HIV disclosure as a unified process, revealing that both avoidance and approach goals coexist. Avoidance delayed disclosure by withholding emotions in conflicted family relationships, yet led to positive outcomes, while approach goals encouraged earlier, open, and factual disclosure in romantic relationships, resulting in better positive outcomes. Greater HIV knowledge, approach goals, and trust encouraged protective behaviors and advocacy. HIV was normalized within the relationship, allowing the couple to manage it alongside everyday uncertainties.

## Introduction

HIV stigma is a significant barrier to ending the AIDS epidemic by 2030 [1]. It hinders HIV testing [2], limits healthcare access and treatment adherence [1], and contributes to non-disclosure [3]. Partner support plays a key role in encouraging behaviors like HIV testing, disclosure, and adherence to Antiretroviral Therapy (ART) [4]. However, most research has focused on how stigma discourages disclosure to partners [5,6], with less emphasis on its effects on serodiscordant couples.

Understanding the impact of HIV stigma on both People Living with HIV (PLWH) and HIV-uninfected individuals is essential, especially in serodiscordant couples. The HIV Stigma Framework highlights how stigma affects both groups differently [7]. HIV-negative individuals often experience stigma as prejudice (negative emotions like disgust, anger, and fear), stereotypes (group-based beliefs about PLWH), and discrimination (behavioral expressions of prejudice). Meanwhile, PLWH face enacted stigma (actual prejudice and discrimination), anticipated stigma (expecting future prejudice and discrimination), and internalized stigma (negative self-perception due to social devaluation).

Self-disclosure is a fundamental aspect of social interaction. HIV disclosure, the verbal sharing of one's HIV-positive status, is encouraged by the WHO and the Joint United Nations Programme on HIV/AIDS (UNAIDS) to enhance health outcomes and foster community engagement for PLWH [8]. HIV disclosure is challenging, as it involves weighing risks, such as rejection and discrimination [9], and benefits, like reduced unprotected sex through better condom negotiation [10], improved ART adherence [11], and psychological well-being [9,12,13]. The Disclosure Processes Model (DPM) by Chaudoir and Fisher [9] offers a framework for understanding the complexities of revealing a concealable stigmatized identity, like HIV. Disclosure decisions are influenced by approach goals (aiming for positive outcomes, like relationship development and intimacy) and avoidance goals (seeking to prevent negative outcomes, like rejection). These are mediated by inhibition alleviation, social support, and changes in social information, fostering intimacy and influencing future interactions [9]. 

Data from a Greek study [14] reveal significant levels of enacted and internalized stigma among PLWH, highlighting the relevance of examining these dynamics in specific cultural contexts: 40% experienced sexual rejection, 71% anticipated it, 15% faced discriminatory reactions from healthcare professionals, 11% were denied services, and 15% had their HIV status disclosed without consent. Additionally, over half internalized feelings of guilt, self-blame, and shame. In response to stigma, 46% isolated themselves from family and friends, 37% avoided sex, and 35% skipped social gatherings. These findings underscore the importance of studying HIV stigma in Greece to address these challenges. This case study aims to explore the personal narratives of a serodiscordant gay couple, focusing on how, when, and why the HIV-positive status was disclosed to the HIV-negative partner and its impact on their relationship dynamics.

## Materials and methods

Participants, recruitment, and ethics

Our research focused on a gay serodiscordant couple, with one partner living with HIV (the discloser) and the other HIV-negative (the confidant). Participants were recruited from a branch of Aretaieion Hospital, with study approval granted under license number 226/18-06-2020 by the hospital's Ethics Committee. In compliance with the British Psychological Society Code of Human Research Ethics, all participants were assured of anonymity.

Data collection

Data collection took place in July 2021 through semi-structured interviews, lasting about an hour, conducted individually with each participant. These interviews were audio-recorded, transcribed verbatim, and initially analyzed in Greek, with relevant sections later translated into English. The interview process was flexible, with questions tailored to the context, and emerging issues explored further. Participants also completed a demographic questionnaire (Table 1). The study’s purpose was clearly explained, pseudonyms were used for anonymity, and participants provided written consent, understanding their right to withdraw at any point, except during report writing.

**Table 1:** Samples’ Demographic Characteristics

Pseudonym	Age	Education Level	Marital Status	HIV Status	Years Living With HIV	Duration of Relationship
Nick	33	Bachelor's Degree	Married	Positive	13	2 years
Peter	43	Bachelor's Degree	Married	Negative	-	2 years

Data analysis

We employed Interpretative Phenomenological Analysis (IPA), an idiographic qualitative approach that explores how individuals make sense of significant life experiences [15,16]. IPA is ideal for examining complex phenomena like health, illness, and identity, combining three core elements: phenomenology (first-person lived experiences), hermeneutics (interpreting deeper meanings through a double hermeneutic process, where the researcher interprets participants’ interpretations), and idiography (in-depth, context-specific analysis of unique individual experiences). 

IPA involves six steps: (1) immersing in the data through repeated reading to grasp the participant’s experience; (2) taking initial notes, analyzing semantic content, and making detailed, descriptive, linguistic, and conceptual comments - this is the most time-consuming step; (3) developing emergent themes by combining the participant's words with the analyst’s interpretations, reflecting the dynamic interplay between description and interpretation; (4) identifying connections among themes; (5) repeating the process for subsequent cases to allow new themes to emerge; and (6) identifying patterns across cases, presented in a table of themes nested within super-ordinate themes [15,16].

Investigator triangulation

This study used investigator triangulation, with the first three authors conducting separate data analyses before collaborating. Following Denzin's [17,18] work on triangulation and Smith's quality indicators for IPA [11,19], this approach minimized bias and enriched interpretations by integrating diverse perspectives, ensuring a coherent narrative and an experiential account thoroughly grounded in the participants’ words, enhancing the credibility and reliability of the findings.

## Results

The analysis revealed that HIV disclosure is a complex process, shaped by decision-making, stigma experiences, and trust development. It involves navigating fears, managing acceptance, and building strong relational foundations. The analysis identified one superordinate theme with three underlying themes, as shown in Table 2.

**Table 2:** Superordinate Theme: The HIV Disclosure Process

Themes	Explanation
Theme 1 - Navigating HIV Disclosure: Decision-Making and the Disclosure Event	Captures the thoughtful, evolving process of deciding when and how to disclose HIV status, balancing self-protection and emotional openness based on past experiences and the nature of each relationship.
Theme 2 - HIV Acceptance and Stigma: Pathways to Disclosure	Adapting to life with HIV explores the journey from initial fear and uncertainty to resilience and acceptance, as individuals learn to manage the practical and emotional challenges of living with HIV. Disclosure of HIV is facilitated through a journey of education, support, and positive reframing, reducing stigma and embracing HIV as a manageable part of life.
Theme 3 - HIV Disclosure: Building a Foundation of Trust and Navigating Life's Broader Uncertainties	Illustrates how early, honest disclosure fosters a strong foundation of trust and openness in the relationship, allowing the couple to normalize HIV as a manageable part of life, navigated alongside everyday challenges, strengthening the bond without defining it.

Theme 1. Navigating HIV disclosure: decision-making and the disclosure event

Decision-Making

After living with HIV for 13 years, Nick’s decision to disclose his status was shaped by his past experiences and accumulated knowledge. In disclosing to key family members, he employed avoidance-focused goals, anticipating negative reactions due to prior conflicts with his sister, abusive father, and the need to explain avoiding military service to his aunt. While disclosure to his father yielded no benefits or harms, disclosures to his sister and aunt had positive outcomes, strengthening relationships and reducing anticipated stigma. These experiences likely boosted his confidence for future disclosures, highlighting how initial outcomes shape subsequent decisions.

Nick: I told my sister early (one year after diagnosis) because we had a conflicted relationship. We had a terrible relationship, but now, thank God, it's better. (…) I told my aunt later (two years after diagnosis). I had to tell her because I didn’t go to the army because of it. She asked me once, twice, three times. I kept coming up with different excuses, and finally, she said, ‘Get serious and tell me what’s going on’.

Nick: We were outside the hospital while my aunt was dying … Under pressure, I told him (to his father, four years after diagnosis), ‘I’ll tell you. I’m gay and HIV positive.’ He reacted awkwardly and said he should have taken me to a psychologist when I was young. I replied, ‘Obviously, yes, but for other reasons.’

In romantic relationships, Nick’s approach shifts to being more approach-focused, aiming for intimacy and understanding. With his second HIV-negative partner, he pursued positive outcomes, working to alleviate fears and reinforce trust.

Nick: This person (the second HIV-negative partner) made me calm down. He made me trust in our togetherness rather than staying in my fear.

Nick: The truth is, I always want to explain everything to him (the third HIV-negative partner) (...) so that the other person gets deeper into your soul.

In his subsequent relationship, Nick maintained this approach, deepening connections and fostering shared vulnerability. However, his first relationship revealed a less supportive environment at first, with his partner showing some fear, but acceptance later.

Nick: With my first partner, we used condoms, and I felt at times that he was afraid. I tried to justify it. But if you ask me now, if it were my current relationship, I wouldn’t justify it because I wouldn’t put myself in a situation where the other person is afraid, even just a little. If I find someone I like, but I sense from the start that they’re afraid, I don’t give in - I walk away. And that, to me, is healthy. I protect myself.

These reflections highlight Nick’s focus on self-protection, indicating a thoughtful approach to maintaining his well-being in relationships, emphasizing the importance of mutual understanding and support.

The Disclosure Event

Nick’s disclosures varied significantly by relationship type with the confidant. With family, he aimed to protect both their emotions and his own by delaying the disclosure, while with partners, he was direct and factual, typically disclosing by the second date. Nick actively involved partners in his care, including discussions with his physician.

Peter: He (his prior partner) told me he had HIV, and I said it didn’t bother me. I told him (to his current partner) that it didn’t concern me. Of course, he didn’t know about my past, specifically that I have friends who are HIV-positive.

Peter’s prior experiences with HIV-positive individuals allowed him to approach his current partner’s disclosure with calm acceptance, further highlighting the role of experience as a confidant in shaping these interactions.

Theme 2. HIV acceptance and stigma: pathways to disclosure

In the early days after his diagnosis, Nick felt fear and uncertainty about his life expectancy and the risk of transmitting the virus. He also grieved potential losses, such as not having children. However, as he learned about effective treatment, his fear faded, despite the unsettling way his diagnosis was communicated.

Nick: She (the physician) says to me, ‘I want you to stay calm,’ and I ask, ‘Why?’ She says, ‘You are HIV-positive.’ I ask, ‘Am I going to die? She replies, ‘I don’t want you to go and jump out of a window.’ I responded, ‘Am I dying?’ She says, ‘With treatment, you will be fine.’ Then why would I jump out of a window?

Nick’s fears shifted to practical concerns, like medication and lifestyle changes. By taking control of his HIV, he turned uncertainties into opportunities that wouldn’t have arisen otherwise. Financial assistance from HIV-related programs enabled him to support loved ones and pursue higher education, filling gaps in his childhood learning. This coincided with a pivotal moment in his life, marking his emergence into adulthood, where he embraced both newfound freedom and the challenges of living with HIV. Nick’s resilience is evident in his self-efficacy and positive reframing, as he continues to live a normal life despite his diagnosis.

Nick: I was just starting my life back then. During that time, my freedom was emerging - from abuse, institutions - what could possibly stop me, HIV? I had decided to live my freedom. I didn't even know what exactly HIV was. I could not even read it. I wouldn’t let it bring me down or take my life.

Nick: She (his aunt) was upset and worried, and I think she felt responsible for protecting me after that. However, I didn’t let her, and I showed her that I could live like everyone else.

As Nick navigated these challenges, he encountered minimal prejudice or discrimination related to his HIV-positive status. Although one friend and his first partner initially expressed fear, this did not lead to rejection or overt discrimination, indicating low enacted stigma. These experiences suggest that any initial stigma was minor and not deeply rooted. Despite anticipating negative reactions, Nick’s disclosure to his sister, and later to his aunt, resulted in improved relationships, further reducing his already low anticipated stigma. Nick experienced no stigma from subsequent partners or significant relationships.

Nick: With my current partner, similarly, there was no problem at all. In general, I think I’ve been lucky in this aspect.

Nick: He even suggested we have sex without a condom. He was already informed that as long as I’m taking my medication, there’s no risk of transmission. He was less afraid than I was (…) I felt that I wasn’t ready to make such a move.

Interviewer: What was it that held you back?

Nick: Fear. And the need to protect the other person (…) but this person made me calm down. He made me trust the ‘togetherness’ rather than staying in my fear.

Regarding internalized stigma, Nick initially felt fear and guilt when his second partner suggested condomless sex. However, his growing comfort and trust in the relationship indicated that this stigma was not overwhelming. Education, communication, and his partner’s support significantly reduced his internalized stigma, showing that it was present but managed through their informed and positive approach. While traces of stigma exist, they don’t dominate his self-perception. He focuses on survival and positive reframing, refusing to let HIV destroy or depress him.

Nick: I also did group therapy, but there I really realized that I don’t belong in that group (…) Seeing how other people handled it gave me strength because it was an example of what not to become.

Interviewer: What do you mean by that?

Nick: Depressed because of HIV (…) If you experience it negatively, you don’t live; if you experience it neutrally, you’ll survive. If you experience it positively, you’re schizophrenic (he laughs).

Peter’s lack of prejudice and fear toward HIV stems from his informed understanding of the virus. His interactions with PLWH are marked by ease and acceptance, with his positive response to early disclosures reflecting confidence in managing the situation - a perspective shaped by past experiences.

Peter: I’m not afraid of HIV. I’m afraid of a thousand other things I could get if I don’t use a condom. The last thing I would fear would be HIV.

Peter: An illness, like other illnesses, doesn’t mean anything special. It doesn’t worry me more than some other diseases (…) I believe that everyone gradually adjusts and gets used to living with it. I don’t think it has shown any significant changes in their lives practically, meaning it hasn’t hindered them in anything - not in their social circles, not in their travels, not in their work, or anywhere.

Peter rejects stereotypes that portray HIV as uniquely negative or stigmatizing; instead, he normalizes it as a manageable illness that neither defines a person’s life nor restricts their activities or relationships. His approach, characterized by support and acceptance, fosters an inclusive environment for PLWH and further facilitates disclosure.

Theme 3. HIV disclosure: building a foundation of trust and navigating life’s broader uncertainties

Building a Foundation of Trust

Early disclosure set a strong foundation for openness and trust, shaping how each partner perceived the other’s character. Nick’s decision to disclose his HIV status early and involve Peter in his medical care underscored his commitment to honesty and transparency, fostering a deeper connection and providing reassurance for Peter.

Peter: Very honest and right to me; I would have wanted to know early on. In terms of honesty, this is important - you make decisions with a clear picture. It positively influenced me. Okay, we’re on the right track; let’s move forward. This information brought me closer to him rather than pushing me away (…) Our relationship is just a normal relationship. HIV doesn’t affect the relationship at all.

Peter’s positive reception of the disclosure strengthened their bond, enabling open communication and establishing trust as a core element of their relationship. HIV is normalized within their partnership, viewed as a manageable aspect of life that does not define or disrupt their connection. Instead, it acts as a catalyst for deeper trust and mutual understanding.

Navigating Life’s Broader Uncertainties

HIV is a part of their lives, but it doesn’t define their relationship. Their focus remains on everyday challenges and uncertainties common to all couples, with HIV being just another aspect they manage together.

Nick: We have many disagreements. We react differently to things, and these different reactions cause explosions. We try very hard to grow, and each of us tries to control himself. We have a great time, we have dreams, we share common interests.

Peter: The relationship has the usual problems that every relationship has - what one person wants or doesn’t want, can or can’t do, and we must find the golden mean. But HIV is not an obstacle in our relationship.

Their relationship dynamics reflect a process of setting aside HIV to develop positive future plans, effectively managing the uncertainties of HIV so that life need not change because of the diagnosis.

## Discussion

Research on disclosure processes often focuses either on the confidant’s reactions [20] or the discloser’s role [12]. Some studies [21] examine factors influencing the decision to disclose, such as goals and anticipated reactions, while others focus on outcomes [22], leaving gaps in understanding the full process. Our case study addresses these gaps by viewing HIV disclosure as a unified process. Through the personal narratives of a serodiscordant gay couple, we explore the motivations and relational impact of the discloser’s decision to share his HIV status.

The decision-making process in HIV disclosure is driven by two motivational systems [9,21,23,24]: approach and avoidance goals. Approach goals focus on potential positive outcomes [9,23,24], where individuals aim to close the gap between themselves and their goals by focusing on positive stimuli [24]. In contrast, avoidance goals focus on potential negative outcomes, driving individuals to move away from negative states [24]. These systems shape how individuals perceive and react to their environments. Evangeli and Wroe [13], in their systematic review, found that HIV disclosure anxiety often stems from fears of negative outcomes and hypothesized that this anxiety may vary depending on the confidant, as many studies did not specify the disclosure recipient.

In our study, both avoidance and approach goals emerged in the same individual, depending on the relationship. Avoidance goals were common in family settings, where emotional conflict occurred and efforts were made to conceal the HIV-positive identity. In contrast, approach goals were seen in romantic relationships, with brief, factual disclosures aimed at fostering intimacy. Approach goals also involved HIV-negative partners in care, including discussions with the physician. Timing differed: with avoidance goals, disclosure was delayed (one to four years), while with approach goals, it occurred by the second date.

While all concealable stigmatized identities face social devaluation, the extent varies based on perceived controllability or risk to others, shaping the disclosure process. The DPM suggests that those with highly stigmatized identities, like HIV, often adopt avoidance goals due to fear of stereotypes and rejection, which reduces disclosure benefits and increases vulnerability to negative outcomes. While avoidance may offer short-term relief by ending information suppression, it can limit long-term benefits by maintaining a focus on rejection and stigma [9]. Our case study supports and expands the DPM by showing that, even with low levels of enacted, anticipated, and internalized stigma, avoidance goals were used when the confidant was a family member. Notably, the discloser experienced no harm from using avoidance in conflicted relationships. In fact, disclosure strengthened his relationship with his sister and deepened the positive bond with his aunt, but yielded minimal benefits with his abusive father. This highlights the critical role of relationship dynamics and stigma in shaping disclosure outcomes.

Research shows that self-acceptance, by reducing internalized HIV stigma, improves comfort with disclosure and promotes HIV prevention advocacy [25]. Our study supports this, suggesting that low levels of stigma, combined with approach or avoidance goals and positive or neutral outcomes, facilitated disclosure. We found that when the discloser had approach goals and greater HIV knowledge, he was more likely to engage in protective behaviors and advocacy, such as involving HIV-negative partners in care by introducing them to his physician. HIV advocacy was absent with avoidance goals in conflicted relationships.

Disclosure is a dyadic process impacting both the discloser and the confidant, yet few studies explore this [9]. In our case study, the discloser revealed his status to HIV-negative partners, facing initial fear from the first partner but eventual acceptance, and with positive outcomes and acceptance from the rest. Disclosure to his current partner was facilitated by his partner’s prior experience as a confidant for PWLH. The absence of stigma and greater HIV knowledge enabled comfortable acceptance. The DPM [9] views disclosure as an ongoing stigma management process, shaped by feedback loops. Our findings support this, highlighting the role of personal character, resilience, and positive reframing in managing stigma. Self-disclosure fosters intimacy in relationships [26], while partner disclosure and perceived partner responsiveness deepen this connection [27]. Emotional disclosures are more effective than factual ones [28]. In our case study, factors such as relationship type, conflict levels, low stigma, and disclosure goals (avoidance or approach) shaped intimacy. Emotional disclosure with avoidance goals deepened intimacy in moderately conflicted family relationships but had no effect in severely conflicted ones. Factual disclosure with approach goals in non-conflicted partner relationships strengthened intimacy, encouraged discussions about unprotected sex, and built trust and decision-making.

Perrett and Biley [29] suggest that adapting to life with HIV involves accepting the diagnosis, broadening one’s perspective, and realizing that 'nothing significant has changed,' allowing for a sense of normalcy. This concept of 'existing as-was' shifts the view of HIV from life-altering to manageable. Our case study supports this, showing HIV as a normalized aspect of the couple’s relationship, which they navigate alongside everyday challenges, without disruption to their bond.

Limitations

This case study has several limitations, primarily those inherent to qualitative research, such as the inability to demonstrate causality and directionality. Our findings are not generalizable to heterosexual or even other homosexual seroconcordant or serodiscordant couples. Like all case studies, our findings show the existence of a phenomenon, not its prevalence. One unique mediator factor in our study was the low stigma experienced by the participants. This low level of stigma shaped the disclosure process, aligning with the subjective and relational experiences described by them. We did not use quantitative tools to measure stigma; instead, we relied on qualitative methods to capture experiential knowledge in Greece - where stigma is typically high - because we wanted the disclosure experience to flow freely and focus on the narrative. This aligns with IPA’s goal of bracketing assumptions. Further qualitative, quantitative, and mixed research is needed to explore current stigma levels in Greece, especially given the unexpectedly low stigma in this case study.

## Conclusions

Our study sheds light on HIV disclosure as a dynamic process influenced by avoidance and approach goals, shaped by the relationship context. By examining the narratives of a gay serodiscordant couple, we show how relationship type, stigma levels, individual experiences with HIV disclosure, and motivations impact disclosure. These findings highlight the importance of tailored interventions, fostering open communication, and addressing both emotional and factual aspects of disclosure. Reducing stigma is crucial for promoting healthier disclosure and HIV advocacy, offering valuable insights for improving support systems.
